# Analyzing sales of the Korean restaurant franchise during the COVID-19 pandemic with the mixed-effects model approach

**DOI:** 10.1371/journal.pone.0293147

**Published:** 2023-10-18

**Authors:** Changro Lee, Youngmo Koo

**Affiliations:** Department of Real Estate, Kangwon National University, Chuncheon, Gangwon-do, Republic of Korea; The University of Hong Kong, HONG KONG

## Abstract

Using point-of-sales (POS) data, the sales trends of 48 member stores of a Korean restaurant franchise during the COVID-19 pandemic were analyzed. As daily sales are nested in each member store of a franchise, the hierarchical structure of POS data was fully and effectively utilized by employing a mixed-effects model. The results showed that although sales volumes in all member stores were negatively affected by the pandemic, the level of impact varied according to store location: sales at some stores were drastically reduced, while a few others even achieved a slight increase in sales during the pandemic. These findings suggest that the government support policy for small business owners should be designed in a locally optimized way, to take account of neighborhood characteristics and the degree of sales loss for individual business owners.

## 1. Introduction

Point-of-sales (POS) data are transaction-level records retrieved and collated from sales stores; these data are used extensively and effectively by companies to improve their business performance. By analyzing POS data, inventory can be managed more accurately to avoid frequent stockouts. In addition, the analysis of POS data can help managers anticipate and identify peak sales periods, thereby allowing them to formulate an optimal strategy for resource allocation. The data can also help business owners focus on promotions for products that have not sold well in the past.

Almost all industries have been affected by the COVID-19 pandemic over the last three years, and franchise businesses in the retail sector are no exception. The investigation of POS data clearly shows that sales volumes in franchise stores reduced sharply during the pandemic. To stimulate the economy and compensate for the loss of sales by small business owners, the government of South Korea has enforced various subsidy policies, such as the reimbursement of a maximum of 75% of the labor cost and cash/voucher-coupon aid [1].

This study analyzes POS data in a Korean franchise business during the COVID-19 pandemic and discusses how the pandemic affected the sales performance of franchise member stores in South Korea. A mixed-effects model is employed to efficiently capture the nested structure of the POS data. The results are interpreted from both temporal and spatial perspectives, and implications for government policy are provided.

Several previous studies have analyzed and forecast sales volumes using POS data, but mainly through the temporal evaluation of sales trends. However, this study also attempts to investigate sales changes in a franchise business from a spatial perspective, because member stores tend to be spread out geographically within countries. In addition, unique circumstances like those engendered by the COVID-19 pandemic are worth separate examination; providing valuable direction to government policies during severely depressed periods. Finally, we attempt to fully utilize the nested structure of POS data to identify sales patterns in each member store in a more granular manner.

The remainder of this paper is organized as follows. Section 2 reviews the POS data and presents a relevant approach for analysis. Section 3 describes the dataset, study area, and model specifications. The results and relevant implications are presented in Section 4. Finally, a summary is provided and directions for future studies are explained in Section 5.

## 2. Literature review

### 2.1 Analysis of point-of-sales data

POS data refer to information collected from a store’s payment system. POS data indicates the point at which corporate data collection commences, and provides the foundation for any subsequent data collected by a company. The data play a critical role in various business processes such as inventory management, sales forecasting, and analysis of customers’ purchasing behaviors. In the era of big data, POS systems provide an integrated data collection tool for the seller: vast amounts of granular POS data indexed by customer IDs are generated in real time and readily available for inventory management and sales prediction [2].

Therefore, it is not surprising that industry stakeholders and scholars have attempted to leverage POS data in business analysis. Chen et al. [3] examined POS data from convenience stores to forecast sales using the moving average method. Fukuhara et al. [4] similarly investigated POS data in Japanese restaurants and demonstrated that restaurant service processes can be improved through the analysis of both POS data and their visualizations. Williams et al. [5] forecast product orders in grocery retailers using an exponential smoothing method equipped with a vector error correction process. Regression models and their advanced variants, such as time-series models, are typical approaches for analyzing future demand when POS data are used as explanatory variables [6–8].

With the rising popularity of artificial intelligence, machine learning algorithms are increasingly being applied to POS data. Sexton and Hignite [9] predicted daily sales in a clothing franchise business by using a neural network. Yang and Sutrisno [10] used the number of sales in the first few business hours and forecast sales in a bakery franchise business for the remaining hours in a single day because of the short shelf life and perishability of the product. Their forecasting tool was also a neural network. Machine learning algorithms other than neural networks have also been employed in the literature. Antipov and Pokryshevskaya [11] used a gradient boosting algorithm to predict the sales of convenience store foods such as pretzels and cereals, and Yennimar et al. [12] employed a random forest algorithm to predict vitamin sales in drug stores during the COVID-19 pandemic.

POS data are structured data; that is, traditional tabular data formatted with rows and columns. Recently, unstructured data, such as free-form texts, have been utilized in addition to POS data to enhance the predictive accuracy of the sales-prediction model. For example, Zhu [13] used both the sales dataset and comments on products by customers (online reviews) to improve conventional sales forecasting models. He used long short-term memory and convolutional neural networks to process consumer comments. Unstructured data have been difficult to analyze until recently, as there were no suitable techniques to process this type of dataset. However, with support from deep learning algorithms like neural networks, free-form texts, including customer reviews and news articles, have become amenable to analysis.

Previous studies have tended to focus on temporal patterns of sales, such as historical trends and seasonality. As a franchise business generally operates several member stores located nationwide, geographical sales patterns are also worthy of investigation. However, most prior studies lack a spatial perspective when analyzing the sales patterns of member stores.

Due to the social distancing and lockdown measures induced by the pandemic, almost all businesses in cities experienced a dramatic decline in their sales volumes [14,15], or changes in business operating models such as fintech-based lending [16]. The pandemic also caused large-scale logistics disruptions in rural regions, which exacerbated the reduction in sales [17]. In addition to these tangible effects, intangible effects such as distress and anxiety contributed to a sharp decrease in business performance [18]. However, there are few studies on business performance during severely depressed periods such as the COVID-19 pandemic. An exception is Yennimar et al.’s study [12], but their approach is time-oriented and thus lacks investigation of the spatial distribution of sales volumes. Finally, most previous studies did not pay attention to the nested structure of POS data and failed to fully utilize the nesting relationship between daily sales and stores; the daily sales values from the same store are likely to be more correlated to each other than the daily sales values from other stores, characterizing POS data as nested. In this case, a mixed-effects model is ideal.

### 2.2 Mixed-effects model

A mixed-effects model is an extension of a simple regression model that allows for both fixed and random effects (hence the name “mixed-effects”). When multiple levels exist in the dataset, such as students sampled from within classrooms, variability in the outcomes, such as exam scores, can be interpreted as being either within or between classrooms. Exam scores at the student level are not independent because students in a given classroom tend to exhibit similar performances.

A few solutions have been suggested to process data with such a nested structure: aggregating the individual observations within the same group and fitting a single global model to the averaged dataset; separating data for each group and fitting a model to the separated dataset repeatedly; fitting a single model to the original dataset but employing the group factor as a categorical explanatory variable. However, these alternatives inevitably discard part of the information in the nested dataset during analysis. Therefore, a mixed-effects model is an ideal tool to mitigate these issues.

Student performance varies according to individual characteristics and school-level environments. Thus, a mixed-effects model that accounts for a hierarchical data structure with students nested in schools is a popular approach for analyzing student performance [19–21]. Similarly, the performance of hospitals and doctors has been frequently evaluated using a mixed-effects model because patients are intrinsically grouped by where they are hospitalized [22–24].

Although POS data in a franchise business essentially have a nested structure, there have been few attempts to investigate daily sales from a hierarchical perspective, that is, the daily sales and store levels where sales are realized. This study employs a mixed-effects model to analyze daily sales, while fully leveraging the nested structure of POS data.

## 3. Dataset and model specification

### 3.1 Dataset and study area

POS data from a Korean restaurant franchise were used in the analysis. This franchise specializes in traditional Korean foods such as sausages made of noodle bits and rice. The analysis period was between January 2018 and December 2021; the duration was exactly four years (1,461 days). In South Korea, COVID-19 cases started to spread nationwide from January 2020 [25], which is the exact midpoint of the analysis period. The *Capital Region* (Seoul and the neighboring area around it) was chosen as the study area. Although the franchise operates member stores nationwide, most (48 stores) are densely located in the capital region.

**Fig 1:**
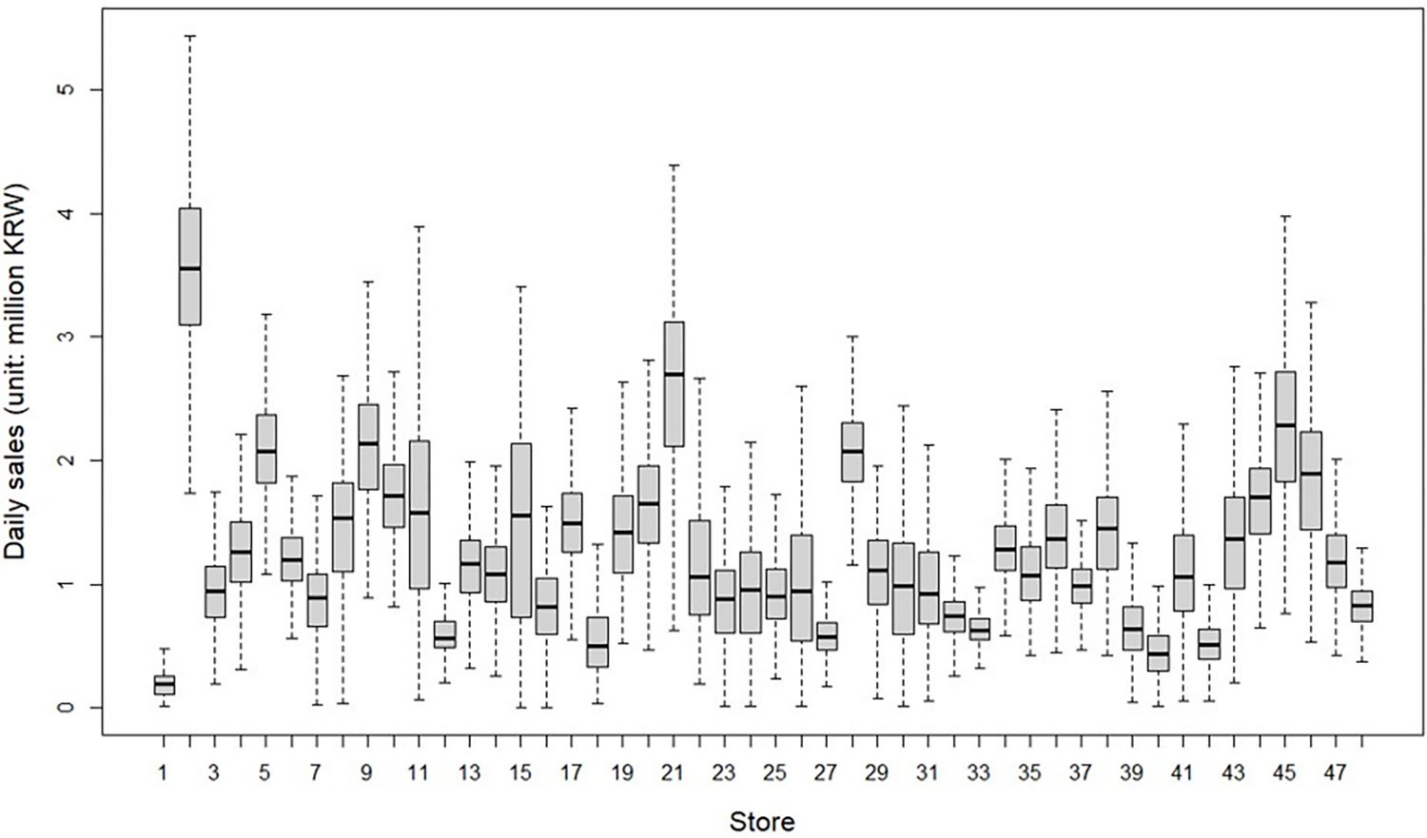
Sales distributions of the 48 stores. Note: Outliers were not drawn for better readability.

**Fig 2:**
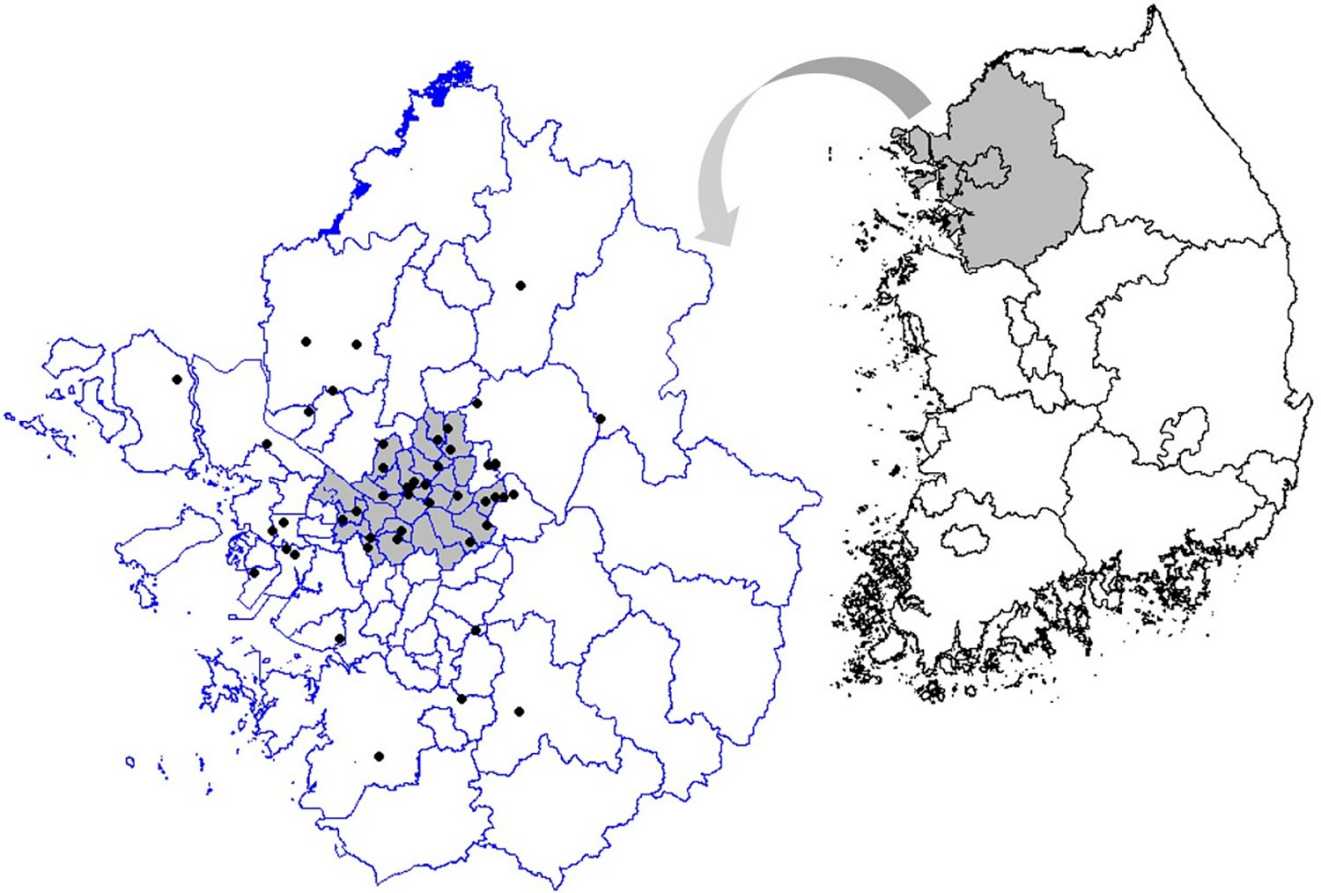
Study area and location of the 48 stores (Figure created by the authors).

A total of 63,970 daily sales were used for analysis. Fig 1 presents the daily sales distributions of the 48 member stores during the analysis period. The magnitude of daily sales varies substantially depending on the store, ranging from less than one million KRW to more than four million KRW. As shown in the figure, the variability in daily sales among stores warrants the use of the mixed-effects model in this study. Fig 2 presents the locations of 48 stores in the capital region. The gray area in the inset map in the figure indicates Seoul, where most stores are concentrated.

An estimation model of business performance could be applied to each of the 48 stores separately, but the observations for a single store are so small that deriving a reliable estimation of sales volume is infeasible. Alternatively, the estimation model could also be applied to the entire dataset without considering the nested structure of the POS data; however, this would lead to inefficient use of the information contained in the dataset, thereby yielding unreliable predictions. In this context, daily sales can be efficiently estimated by considering the effects of the group variable (store) to which daily sales belong, using a mixed-effects model. Details related to a mixed-effects model are succinctly explained in Zuur et al. [26].

### 3.2 Specification of a mixed-effects model

The model is specified in the following equation:

y=Xβ+Zu+ε (1)

Where ***y*** is a 63,970 × 1 column vector (daily sales); ***X*** is a 63,970 × 3 matrix of the three explanatory variables (a fixed intercept, time indexed through 1 to 1,461, and square of the time); ***β*** is a 3 × 1 column vector of the fixed-effects coefficients corresponding to the three explanatory variables; ***Z*** is a 63,970 × 96 matrix for the two random effects (a random intercept and coefficient of the time) and 48 stores, and thus 96 (2 × 48) columns; ***u*** is a 96 × 1 vector of the two random effects (the random complement to the fixed ***β***) for 48 stores; and ***ε*** is a 63,970 × 1 column vector of the residuals. The equation was written following the notations of Winter [27] and Pinheiro and Bates [28].

In other words, the model performs a mixed-effects analysis of the relationship between daily sales and time flow. Time and squared time were entered into the model as fixed effects to capture the nonlinear effects of the passage of time, as below:

$$Daily\;sales=Time+Time^{2}+\varepsilon ,\varepsilon ∼N(0,\sigma^{2})$$ (2)

The intercepts for 48 stores and by-store random slopes for the effect of time were used as random effects. The error term variance is expressed as

$$\alpha_{store}∼N(0,\Sigma_{2\times 2})$$ (3)

This model is often referred to as the *random slope model* in the literature. The model specified in Eq (1)–(3) was determined as the final model by likelihood ratio tests of the full model with the effects described above against the reduced models without the effects in question.

## 4. Results and discussion

### 4.1 Results

The mixed-effects model described above was fitted to the data via the restricted maximum likelihood method, and Table 1 shows the results. To review the validity of the results, data sub-setting and refitting were undertaken. The used dataset was randomly split into four parts of equal size (approximately 16,000 samples), and a mixed-effects model with the specifications from Eq (1)–(3) was fitted. This procedure is similar to bootstrapping in statistics and is frequently used for evaluating the robustness of a quantitative model. The results from each subset were almost identical to those in Table 1, allowing the model adopted by this study to be considered robust and safe for subsequent analyses.

**Table 1:** Results of a mixed-effects model.

Fixed effects (n = 63,970)			
	Estimate	Standard error	t-value
Intercept	1.418	0.098	14.467
Time	-0.025	0.038	-0.653
Time^2^	-0.227	0.010	-22.636
Random effects (group = 48)			
Store level		Variance	Standard deviation
	Intercept	0.460	0.679
	Time	0.058	0.240
Individual level		0.155	0.394

Among the fixed effects, all variables except *Time* are significant, as shown in the t-value column. However, excluding the main term (*Time*) and including its derived term (*Time*^*2*^) was deemed inappropriate; thus, it was decided to retain both terms in the model. In random effects, the standard deviation at the store level is a measure of how much variability in the response variable (daily sales in million KRW) is caused by stores: the standard deviations of the intercept and *Time* variables are 0.679 and 0.240, respectively. These can be expressed by Eq (3) as follows:

$$\alpha_{store}∼N(0,\left[\begin{array}{cc} 0.679^{2} & -0.051\\ -0.051 & 0.240^{2} \end{array}\right])$$ (4)

The standard deviation at the individual level is 0.394, which is *σ* in Eq (2), indicating the variability that is not explained at the store level. The proportions of the standard deviation of intercept and *Time* at the store level are 52% and 18%, respectively, and only the remaining 30% of the standard deviation is attributed to the individual level. This indicates that employing a mixed-effects model that considers the hierarchical structure of POS data, instead of a single-level regression model, is a suitable approach.

**Fig 3:**
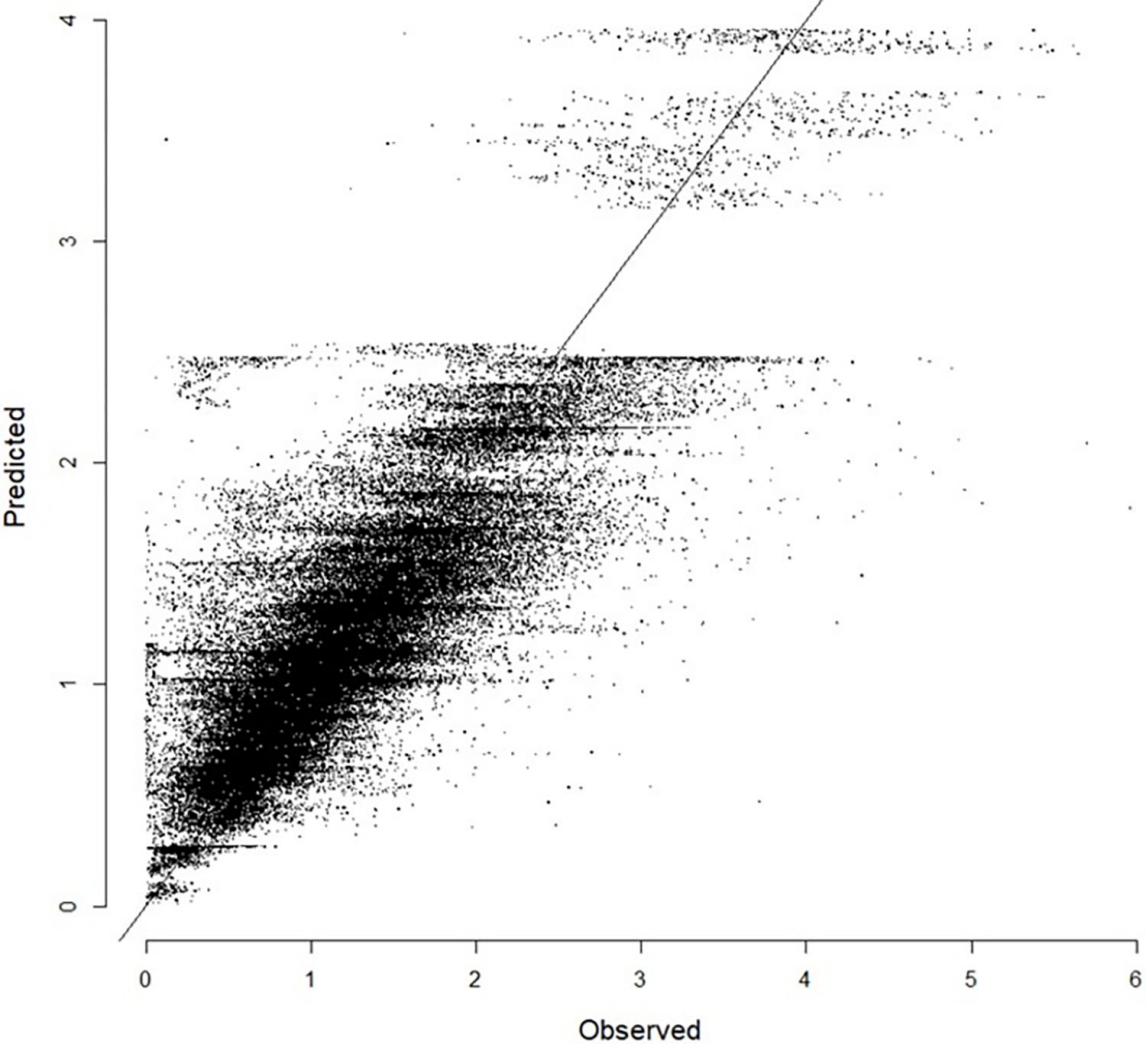
Goodness-of-fit of the model (unit: Million KRW).

Fig 3 shows the goodness-of-fit of the model. The model appears to have difficulty predicting daily sales in a specific range (particularly between two and a half and three million KRW). This is because of the lack of relevant explanatory variables. To capture the minute difference in daily sales among stores, explanatory variables other than the passage of time are required, such as the population of the neighborhood and competition intensity between rival franchise stores. The selection of explanatory variables always involves a compromise between theory and data availability. Although this study only utilized the passage of time and variability between stores, an inspection of the figure did not reveal obvious flaws; thus, the model can be deemed safe for subsequent analyses.

**Fig 4:**
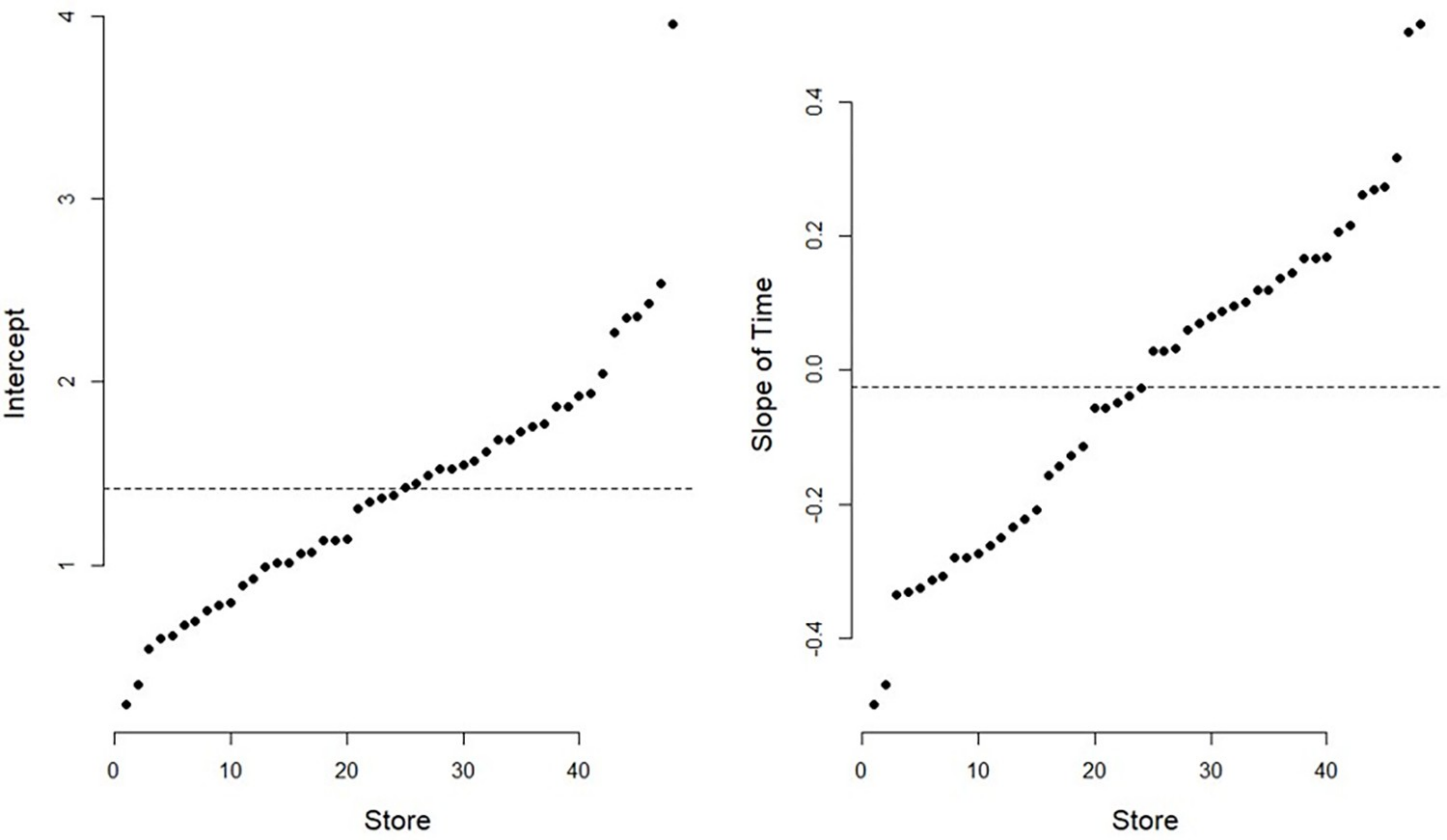
Intercepts and slopes of *Time* for the 48 stores. Note: The horizontal dotted lines represent the intercept (left) and the slope of *Time* (right) in the fixed effects in Table 1, which are 1.418 and -0.025, respectively. Unit in the vertical axis is million KRW.

As a random slope model was utilized through Eq (1)–(3), the 48 stores are allowed to have differing intercepts and different slopes for *Time*. Fig 4 presents the variability of the intercepts (left) and slopes of *Time* (right) for the 48 stores. For example, in the case of intercepts, although the average daily sales (the intercept in fixed effects) is 1.418 million KRW (Table 1), the sales level ranges from less than one million KRW to approximately four million KRW, depending on the store. The variability shown in the figure supports the relevance of employing a mixed-effect model.

### 4.2 Interpretation and policy implications

**Fig 5:**
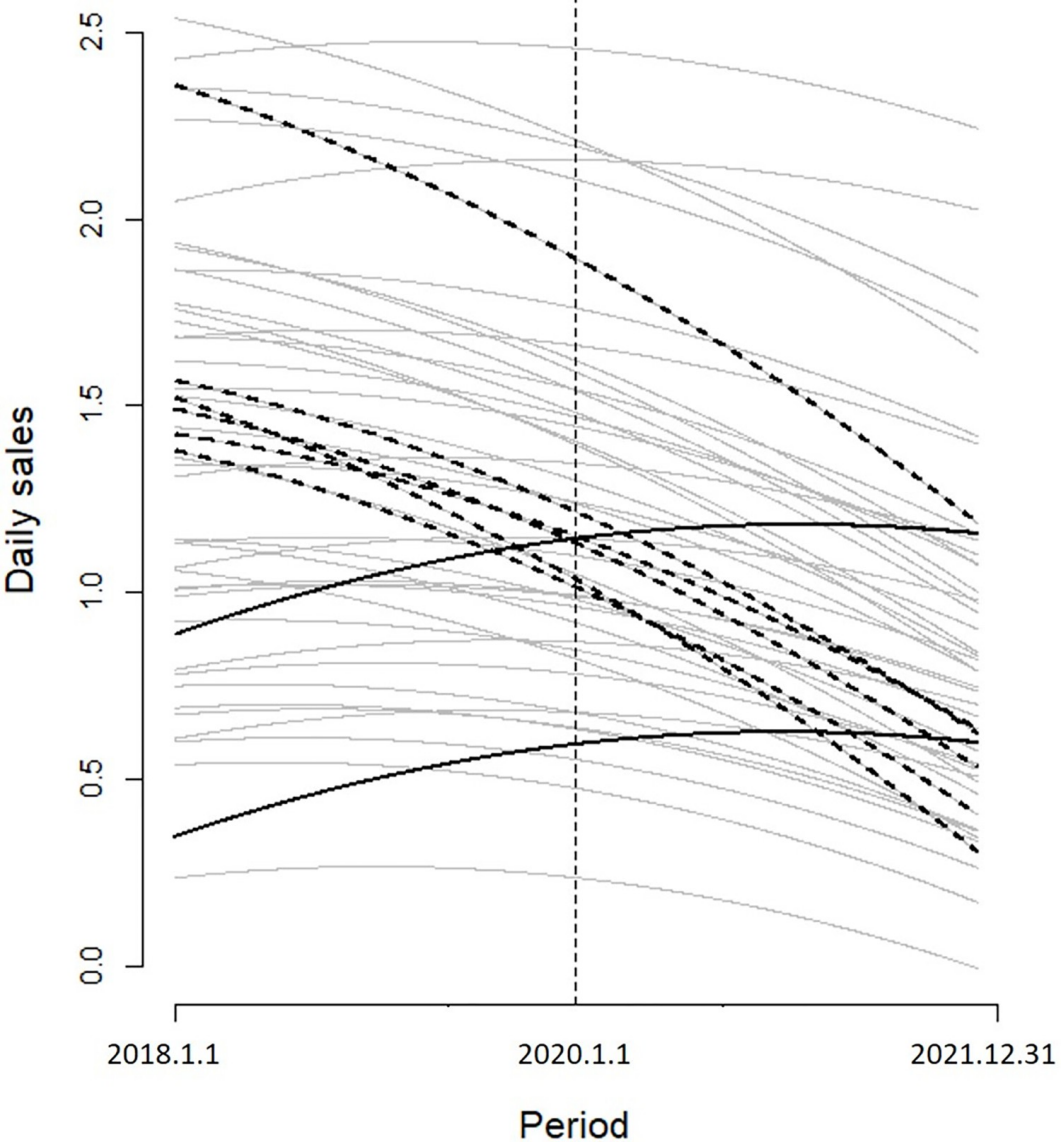
Changes in daily sales in the 48 stores between 2018 and 2021.

Fig 5 shows daily sales trends for each store during the analysis period (2018 to 2021). Each line in the figure was created using random intercepts, random slopes of Time, and a fixed slope of Time2 using indices 1 through 1,461. The vertical dotted line represents the midpoint of the analysis period (January 1, 2020) which corresponds to the outbreak point of the COVID-19 pandemic in South Korea. As shown in the figure, most stores experienced a decline in sales during the analysis period. However, some stores like the six indicated by dotted bold lines, experienced severer sales reductions, while others even demonstrated a slight increase in sales, such as the two indicated by solid bold lines. Fig 5 indicates that the impact of the COVID-19 pandemic on daily sales was not uniform across stores.

**Fig 6:**
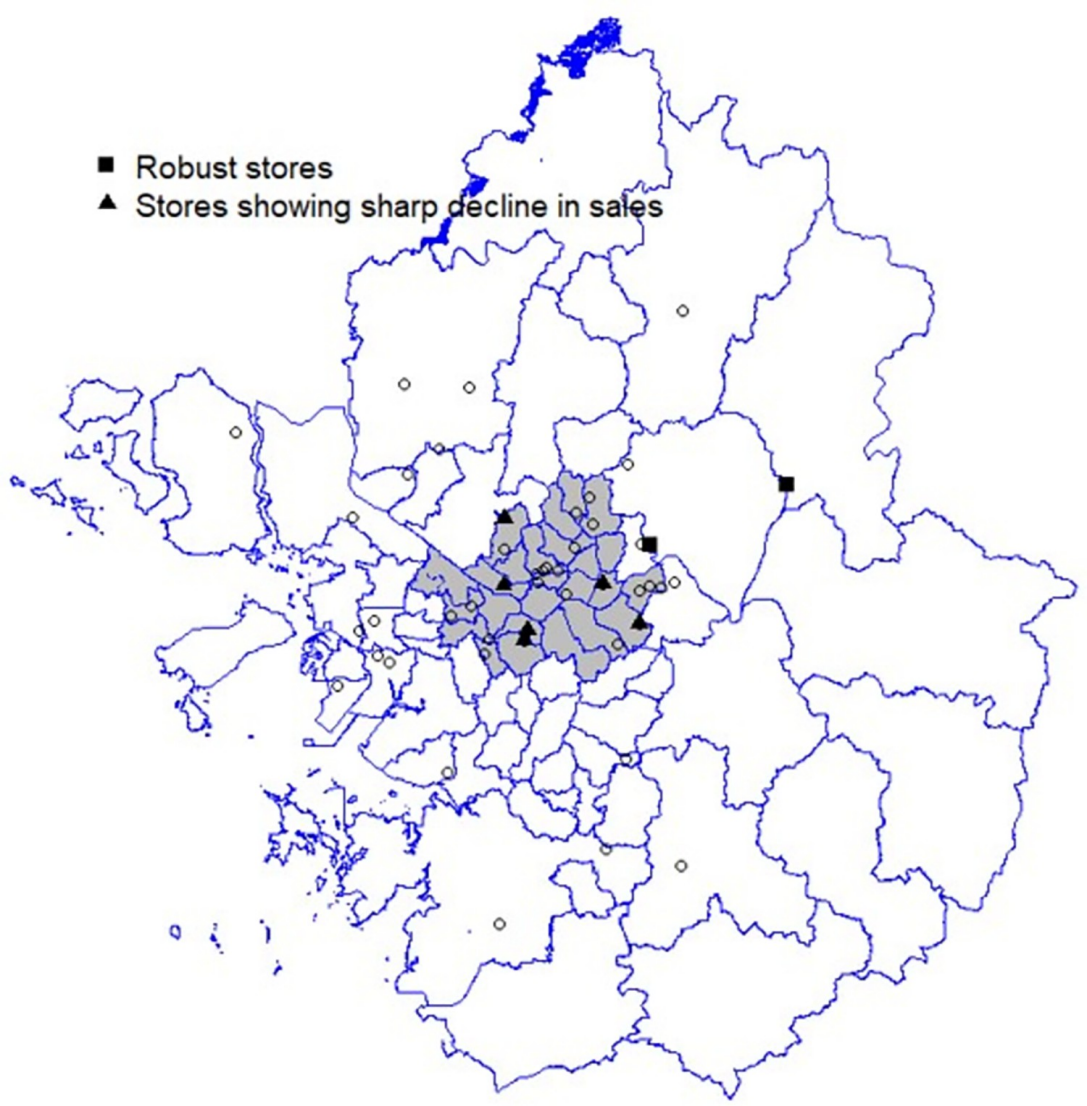
Distinct stores in terms of daily sales changes (Figure created by the authors).

Fig 6 shows six vulnerable and two robust stores in terms of daily sales changes during the pandemic. Of the six vulnerable stores, three are located close to universities: the stores in Sungsil, Hongik, and Seoul National universities. The other three are located close to subway stations: the stores in Ogeum, Gupabal, and Gunja stations. All six stores are in neighborhoods characterized by bustling shopping and entertainment areas packed with fashion brands, bars, and casual eateries. Due to the lockdown policy of the government during the pandemic, sales volumes in these areas were most severely curtailed. In other words, retail stores in shopping and entertainment districts in Seoul were most harshly impacted by the pandemic.

In contrast, two stores located in the eastern part of the study area were unaffected by the pandemic; rather, the stores in Gawoon and Daesung-ri achieved a slight increase in sales volumes. These stores are located in residential areas with dense apartment blocks and single-detached houses. It is well-known that delivery sales increased remarkably since the government enforced the lockdown policy. Thus, in the case of the two stores, it can be inferred that the decrease in in-store sales was offset by an increase in delivery sales.

**Fig 7:**
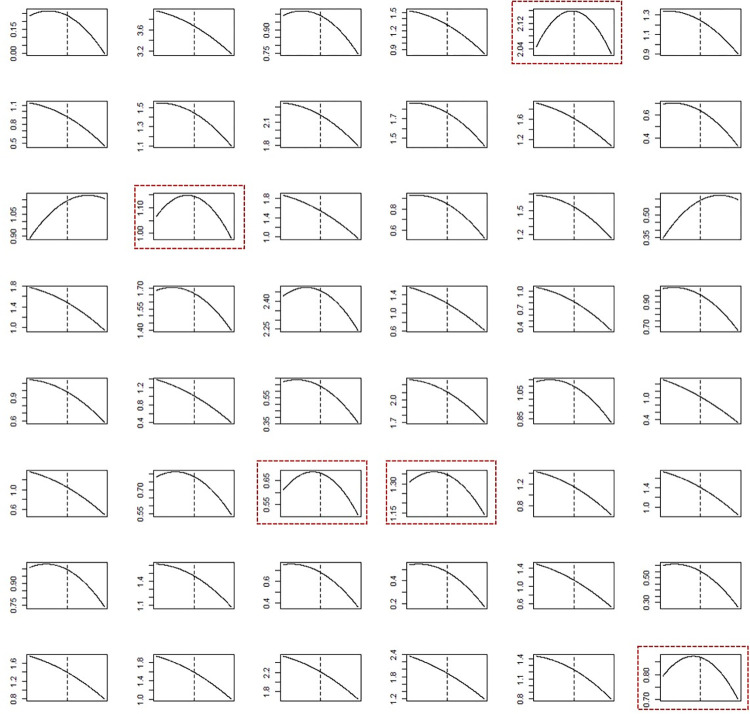
Sales changes in individual stores. Note: Stores indicated in the dotted border line represent stores in Mokdong, Gwangtan, Ganseok, Cheonghak, and Jukjeon, respectively, from the upper left. The vertical dotted line inside each panel represents the midpoint of the analysis period (January 1, 2020).

Fig 7 is an individual version of Fig 5, and presents the daily sales changes for each store. As Fig 5 represents the sales changes of all stores in one graph, it is difficult to discern minute changes in each store, such as the turning point in sales trends. These individual patterns are clearly demonstrated in Fig 7, showing that each store experienced its own trajectory in terms of sales changes. An interesting characteristic is that some stores show an upside-down U-shaped trajectory in their sales changes. These stores are indicated by dotted lines in the figure. They attracted many customers until immediately before the outbreak of the COVID-19 pandemic. However, this increasing sales trend abruptly plummeted after the outbreak. Although additional investigation is required to ascertain accurate reasons for the upside-down U-shape in sales trends in these stores, it is obvious that they are affected most severely by the pandemic.

During the pandemic, the government provided small business owners with various types of support; for example, financial subsidies in cash or as vouchers were provided to the stores affected by the pandemic. However, the subsidy amounts were uniform across the nation. The findings of this study suggest that government subsidies should have been enforced selectively by considering neighborhood characteristics and store sales performance. First, stores in shopping and entertainment districts were found to be most severely affected and thus in need of the most comprehensive support from the government. Second, stores located near apartment blocks and single-detached houses successfully defended their sales performance by compensating for in-store sales losses through online delivery sales. Thus, in the case of such stores, policies for promoting online sales, such as tax deductions for sales arising from electronic transactions should be formulated. Finally, as indicated in Fig 7, the magnitude and pattern of sales losses were found to differ remarkably between individual stores. Some stores experienced extreme sales losses, as shown by the upside-down U-shaped sales loss trajectory in Fig 7. For these stores, inspections by government officials and appropriate compensation packages must be implemented. In short, the government needs to design a locally optimized subsidy policy by considering the characteristics of neighborhoods and the degree of sales loss endured by individual business owners.

## 5. Conclusion

POS data from a Korean franchise business were used to analyze changes in daily sales during the pandemic. The capital region of South Korea was chosen as the study area. A mixed-effects model equipped with varying intercepts and slopes of time for each store was employed. The results revealed that the COVID-19 pandemic disproportionately and differently affected member stores in the franchise business: some suffered higher losses, whereas others demonstrated relatively robust sales performance. These findings imply that the government support policy for small business owners should be designed in a locally optimized manner, considering the location of each business and its neighborhood/trade area characteristics.

This study has a few limitations that deserve mention. First, the 48 member stores are of different sizes and operate different hours: some stores have large floor areas and many staff, but others do not; some stores operate through the whole week, but others run their businesses only on weekdays. Thus, future research should address the lack of explanatory variables related to store characteristics. Second, the findings of this study apply only to the chosen study area, the *Capital Region* in South Korea. Future research should apply the approach adopted in this study to different geographical settings to verify the generalizability of the current findings. Third, this study focused on sales changes during periods of depression, such as the COVID-19 pandemic. Therefore, it is worth applying a mixed-effects model to predict business performance during normal periods. Future research should confirm whether the model used in this study works appropriately across different periods. This will enable a more accurate examination of the changes in business performance, thereby improving the generalizability of the study results.

## Supporting information

S1 File (CSV)
